# Case report: Eyelid symptoms as an early harbinger of staphylococcal scalded skin syndrome: a novel pattern of disease onset

**DOI:** 10.3389/fopht.2025.1689008

**Published:** 2025-12-11

**Authors:** Mario Troisi, Salvatore Troisi, Carolina Mauro, Laura Timpone, Antonella D’Aponte, Ciro Costagliola, Diego Strianese

**Affiliations:** 1 Eye Clinic, Department of Neurosciences, Reproductive and Odontostomatological Sciences, University of Naples Federico II, Naples, Italy; 2 Ophthalmologic Unit, Salerno University Hospital, Salerno, Italy; 3 3Pediatric Unit, Salerno University Hospital, Salerno, Italy

**Keywords:** staphylococcal scalded skin syndrome, pediatric ophthalmology, toxin-mediated keratoconjunctivitis, *Staphylococcus aureus*, exfoliative toxin A/B, desmoglein-1, blepharitis

## Abstract

**Background:**

Staphylococcal scalded skin syndrome (SSSS) is a rare but potentially life- threatening condition caused by exfoliative toxin-producing Staphylococcus aureus. Ocular involvement, although infrequently reported, may lead to vision-threatening complications if not promptly recognized.

**Methods:**

The clinical history, findings, therapy, and outcome of two patients with SSSS exhibiting prominent ocular manifestations at disease onset were analyzed and reported. A comprehensive literature review was performed using PubMed/Medline, Embase, and Scopus incorporating search terms such as “Staphylococcal scalded skin syndrome,” “eye,” “eyelid,” “conjunctival,” “ocular manifestations,” and “ocular adnexal.”

**Results:**

Two pediatric patients with SSSS were identified. Case 1: a 4-year-old boy with initial blepharitis progressing to periocular scaling, conjunctival inflammation, and corneal staining, confirmed as SSSS by clinical findings and microbiology. Case 2: a 38-month-old boy presenting with bilateral eyelid edema, periocular desquamation, and perioral lesions, with negative corneal staining. Both patients were treated with systemic antistaphylococcal antibiotics (including toxin-targeting regimens) and topical ocular therapy (fusidic acid gel and hypochlorous acid spray), resulting in rapid clinical improvement and complete resolution of ocular and cutaneous lesions. The literature review identified a single reported case of a healthy adult with purulent conjunctivitis as an initial manifestation of SSSS.

**Conclusion:**

Although rare, ocular manifestations may serve as an early indicator of SSSS. Prompt ophthalmological evaluation and combined systemic and targeted topical therapy are essential to prevent ocular sequelae. Awareness of this condition among ophthalmologists and pediatricians is critical to prevent complications and potential permanent visual impairment.

## Introduction

Staphylococcal scalded skin syndrome (SSSS) is a severe exfoliative dermatosis caused by toxigenic strains of Staphylococcus aureus, most frequently associated with group II phage (1). The condition is characterized by the acute onset of fever, diffuse erythema, fragile bullae, and widespread desquamation of the superficial epidermis, giving the appearance of extensive burn injuries (2). While it predominantly affects neonates, infants, and young children—owing to their immature renal clearance of bacterial exotoxins—SSSS is rare in adults, where it is associated with significant comorbidities and higher mortality rates (2–4). Differentiation from toxic epidermal necrolysis (TEN) can be clinically challenging due to overlapping cutaneous manifestations, although the level of epidermal cleavage differs between the two conditions. Epidemiological studies report a seasonal pattern, with increased pediatric incidence during summer and autumn months (5, 6).

The pathophysiology of SSSS is driven by exfoliative toxins A and B, which target desmoglein-1, a cadherin-type cell adhesion molecule in desmosomes that anchors keratinocytes in the superficial epidermis. Toxin-mediated cleavage of desmoglein-1 leads to intraepidermal splitting, resulting in loss of skin integrity and subsequent risk of fluid imbalance, thermoregulatory disruption, and secondary infections (7). The toxins, produced at a localized site of infection such as the nasopharynx, conjunctiva, or umbilicus, spread hematogenously to cause widespread epidermal injury. Diagnosis is primarily clinical, often supported by a positive Nikolsky’s sign, and may be confirmed with histopathology or culture from the primary site of infection (8). Prompt systemic antibiotic therapy, supportive care, and close monitoring are essential to reduce morbidity and prevent complications such as sepsis and pneumonia. While mortality in pediatric patients remains below 10% (9, 10), it increases dramatically in adults, reaching 40–63% despite adequate antimicrobial coverage (11).

Although the skin manifestations of SSSS are well recognized, ocular involvement is rarely reported in the literature and remains an underappreciated feature of the disease. The delicate structures of the ocular surface may be particularly susceptible to toxin-mediated epithelial damage, which can result in eyelid erythema and edema, conjunctival hyperemia,superficial keratopathy, and, in severe cases, corneal epithelial defects. These manifestations present a diagnostic and therapeutic challenge, requiring differentiation from primary ocular infections and other inflammatory disorders (12). Delayed recognition may lead to persistent epithelial defects, secondary bacterial keratitis, or long-term visual impairment.

Here, we report two pediatric cases of SSSS presenting with atypical ocular manifestations. Through these cases, we aim to emphasize the importance of early ophthalmic assessment in suspected SSSS, highlight the diagnostic complexities posed by ocular involvement, and discuss therapeutic strategies that can reduce the risk of vision-threatening sequelae.

## Methods

A retrospective analysis was conducted on the clinical history, findings, therapy, and outcome of patients with Staphylococcal Scalded Skin Syndrome (SSSS) exhibiting prominent ocular manifestations at disease onset. The analysis reviewed charts from 2020 to 2025 at an emergency ophthalmology department serving a large population area. A comprehensive literature review was performed using PubMed/Medline, Embase, and Scopus incorporating search terms such as “Staphylococcal scalded skin syndrome,” “eye,” “eyelid,” “conjunctival,” “ocular manifestations,” and “ocular adnexal”, from inception to March 2025.

## Results

Two cases with SSSS exhibiting prominent ocular manifestations at disease onset were identified.

## Case 1

**Figure 1:**
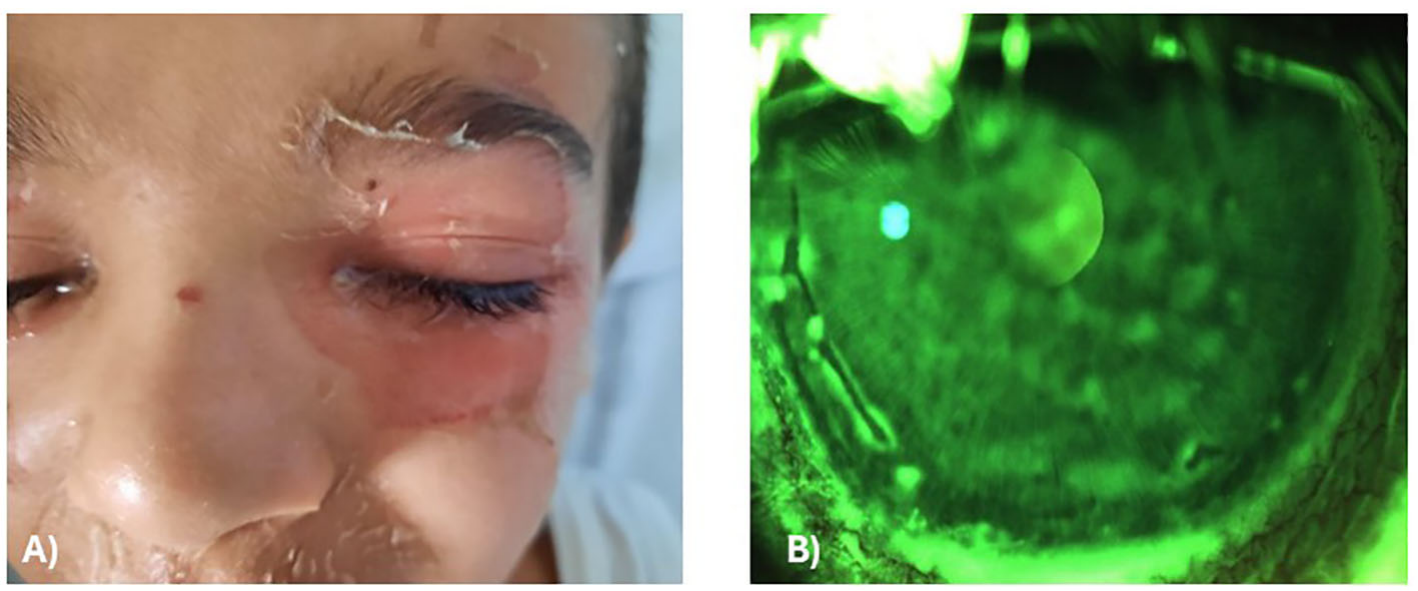
Initial ocular manifestations in the first patient. **(A)** Periocular skin showing marked erythema, edema, and superficial scaling at presentation. **(B)** Slit-lamp anterior segment image of the left eye demonstrating abnormal punctate corneal fluorescein staining, indicative of epithelial involvement.

A 4-year-old Caucasian male presented to the emergency department (ED) with erythematous patches and blisters affecting the face, neck, periocular area, and buttocks. Ocular examination revealed eyelid swelling, periocular scaling, and corneal fluorescein staining (Oxford grade 3) in the left eye, with a negative result in the right eye (**Figure 1**). Fundus examination was unremarkable in both eyes,

The patient had a history of atopic dermatitis, predominantly involving the flexural areas, nasogenial and glabellar regions, and periocular skin, managed intermittently with topical corticosteroids in 7–10-day cycles. He had not received systemic medications in the preceding 30 days and was fully up to date with vaccinations.

One week prior, he developed left eyelid margin inflammation for which Tobramycin 0.3% eye drops (1 drop twice daily) had been prescribed by his pediatrician via teleconsultation. Over the following days, his condition worsened, with periocular pruritus, mucopurulent discharge from the left eye, fatigue, reduced appetite, and the appearance of erythematous, scaly patches on the buttocks. He remained afebrile initially, but gentle rubbing of the periocular skin and antecubital fossa resulted in epidermal detachment with minimal pressure (positiveNikolsky sign). The most intense erythema extended from the left eyelids to the zygomatic area, with milder involvement on the contralateral side. The oral and pharyngeal mucosa were unaffected.

Ophthalmology consultation raised a suspicion of staphylococcal scalded skin syndrome (SSSS), and the patient was referred to the Pediatric Department for further investigation. On admission, conjunctival and exfoliated skin swabs were collected. Laboratory tests revealed a white blood cell count of 9.1 × 10^9/L. The clinical presentation and history supported the diagnosis of SSSS, likely originating from blepharitis of the left eye. The absence of significant mucosal involvement made bullous impetigo and toxic epidermal necrolysis (TEN) less likely.

Initial management included intravenous (IV) hydration and IV nafcillin 40 mg/kg/day in four divided doses. Ocular treatment consisted of fusidic acid 1% gel (every 4 hours) and hypochlorous acid spray (every 8 hours) to the periocular area. Despite treatment, the patient developed fever, generalized desquamation, and worsening periocular inflammation within 12 hours. Paracetamol was given as needed for fever and pain, and buttock lesions were dressed with sterile bandages. By day 2, inflammation and desquamation had progressed to the right periocular area.

Cultures from ocular discharge and erosive skin lesions grew Staphylococcus aureus producing exfoliative toxin B, without toxic shock syndrome toxin-1 (TSST-1) or enterotoxins.

On day 3, with persistent fever and skin lesions, systemic therapy was switched to IV clindamycin 30 mg/kg/day to target toxin production. Within 24–48 hours, the patient became afebrile with marked improvement in pruritus, ocular manifestations, and general condition. Blood cultures remained negative, and leukocyte count decreased to 5.9 × 10^9/L. Skin lesions began crusting and re-epithelializing.

On day 6, IV fluids and clindamycin were discontinued, replaced with adequate oral intake and oral cefixime syrup 8 mg/kg/day. The patient was discharged on day 8 with a 6-day course of oral cephalexin, fusidic acid ocular gel every 6 hours, and hypochlorous acid spray twice daily.

**Figure 2:**
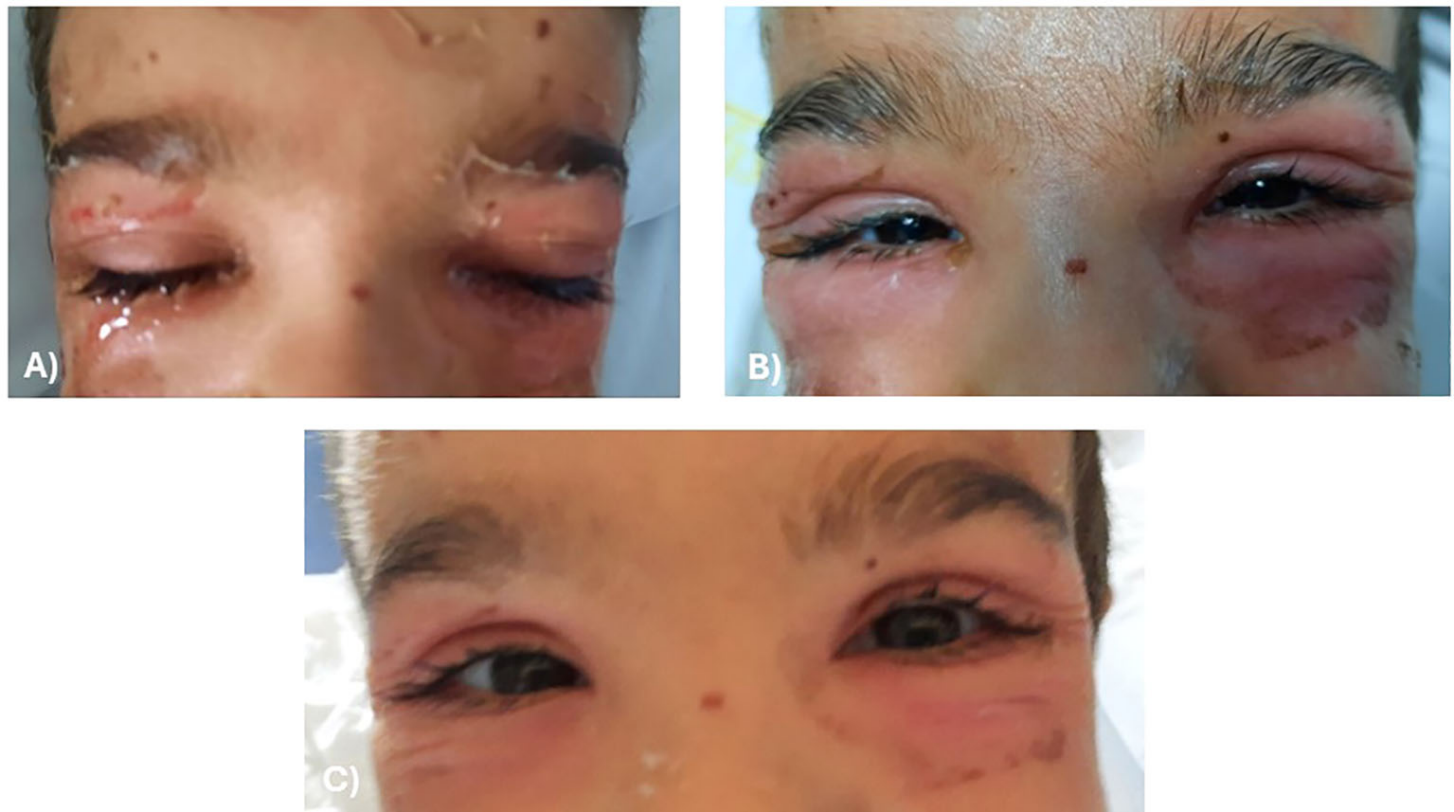
Clinical evolution of Staphylococcal Scalded Skin Syndrome (SSSS) in the first patient. **(A)** Initial erythematous desquamating lesions involving the periorbital region and face. **(B)** Day 3: persistence of lesions despite intravenous nafcillin (40 mg/kg/day in 4 doses); ocular management included fusidic acid 1% gel every 4 h and hypochlorous acid spray every 8 h periocularly. **(C)** Day 6: complete re-epithelialization after targeted systemic antibiotic therapy with intravenous clindamycin (30 mg/kg/day) and supportive topical treatment.

At 6-day follow-up, there was complete resolution of eyelid inflammation and extraocular skin lesions. The clinical course—from initial facial erythema to progressive superficial epidermal detachment and complete re-epithelialization—is shown in **Figure 2**.

## Case 2

**Figure 3:**
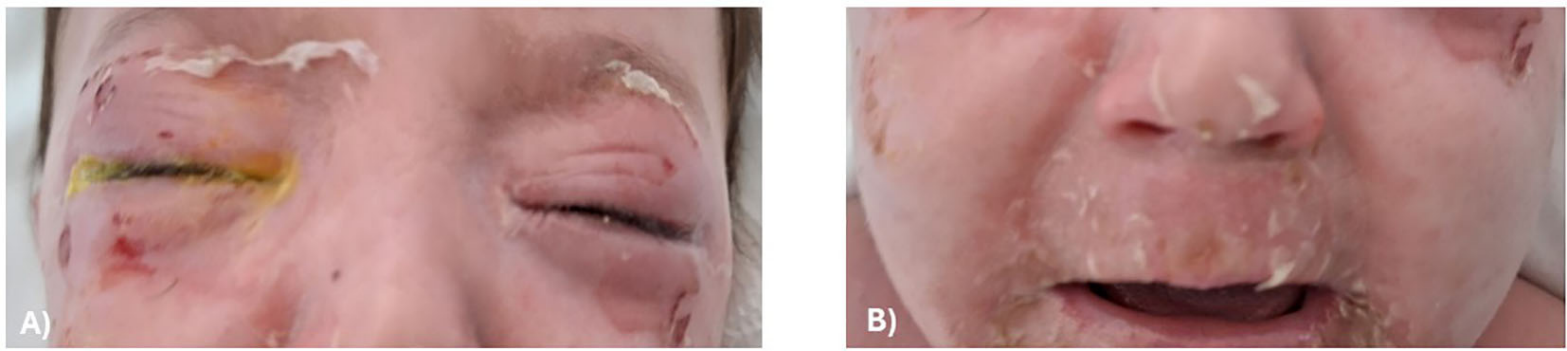
Ocular and perioral manifestations at presentation. **(A)** Bilateral eyelid edema and erythema with superficial epidermal desquamation along the eyebrow region, associated with patient discomfort and difficulty keeping the eyes open. **(B)** Perioral skin involvement with erythematous patches and superficial erosions extending to the vermilion border, accompanied by erosive lesions of the buccal mucosa.

A 38-month-old boy was brought to the pediatric emergency department with erythematous patches and blisters affecting the face, neck, trunk, and buttocks. The lesions were accompanied by pain and hypoergia, and the patient had a low-grade fever (37.8 °C). On ocular examination, there was bilateral eyelid edema and erythema, with difficulty in keeping the eyes open, and evident desquamation of the skin near the eyebrows in both eyes. A corneal fluorescein staining test (fluotest) was performed at the bedside using a binocular ophthalmoscope with a cobalt blue filter after gentle manual eyelid retraction. The result was negative in both eyes, ruling out epithelial corneal defects at presentation. On inspection of the face, erythematous-desquamative lesions similar to those in the periocular region were also noted in the perioral skin, with extension to the buccal mucosa (**Figure 3**). Posterior segment evaluation was entirely unremarkable in both eyes.

The patient’s past medical history was unremarkable for ocular disease or dermatological conditions. There had been no exposure to new systemic or topical medications in the preceding three months. The only medication administered before presentation was paracetamol, given during the two days prior to admission for fever and discomfort, with no prior history of adverse reactions to this drug. The parents reported that two days earlier the child had developed a dry cough, fever, rhinorrhea, nasal congestion, and marked asthenia. These symptoms were initially attributed to viral rhinopharyngitis, particularly given the presence of several cases of influenza and parainfluenza at the nursery school he attended. The subsequent onset of facial and eyelid skin changes alarmed the parents and prompted immediate presentation to the pediatric emergency service.

During the physical examination, gentle rubbing of the perinasal and perioral skin induced epidermal detachment, confirming a positive Nikolsky sign. The pediatric assessment revealed the involvement of the oral and pharyngeal mucous membranes. Based on the clinical findings, a presumptive diagnosis of staphylococcal scalded skin syndrome (SSSS) was formulated during a multidisciplinary consultation between the ophthalmologist and pediatrician, and the child was admitted for further diagnostic work-up and management.

At the time of admission, laboratory testing revealed a white blood cell (WBC) count of 8.5 × 10^9/L. Microbiological samples were obtained from multiple sites, including exfoliated skin lesions, external ear canal, conjunctival sacs, and the nasopharynx, to identify the causative pathogen. A skin biopsy was not performed, both because the clinical presentation was highly suggestive of SSSS and due to the lack of parental consent for an invasive diagnostic procedure at that time.

Empirical systemic antibiotic therapy with intravenous (IV) teicoplanin at a dosage of 10 mg/kg/day was initiated, alongside IV fluid supplementation to maintain adequate hydration. Paracetamol was administered as required for analgesia and antipyresis. Ocular treatment consisted of fusidic acid 1% gel (1 drop, five times daily) and a hypochlorous acid spray applied to the periocular skin three times daily.

**Figure 4:**
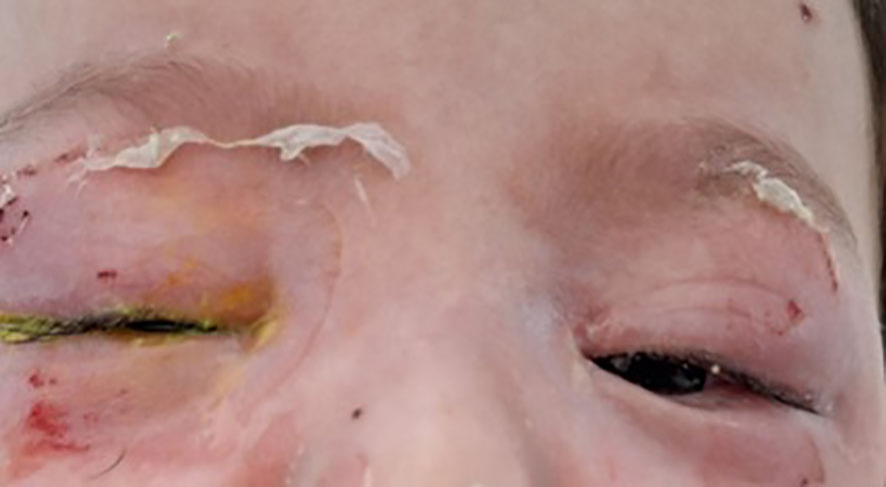
Clinical picture at 48 hours showing marked improvement in eyelid manifestations, managed with fusidic acid gel (1 drop, 5 times/day) and hypochlorous acid spray (3 times/day).

Within 48 hours of initiating therapy, the patient’s clinical condition improved significantly, with complete resolution of fever, marked reduction in nasopharyngeal inflammation, and visible improvement in periocular erythema and eyelid edema (**Figure 4**).

Culture results became available at this stage, confirming methicillin-resistant Staphylococcus aureus (MRSA) growth from all tested sites (skin, ear, conjunctiva, and nasopharynx). The antibiogram revealed susceptibility to both teicoplanin and fusidic acid, supporting the continuation of the current systemic and topical antimicrobial regimen.

Treatment with IV teicoplanin and ocular fusidic acid was continued for six days, during which the patient’s WBC count normalized and both cutaneous and ocular manifestations progressively resolved. No new lesions appeared, and existing erosions underwent re-epithelialization. Given the favorable course, the patient was discharged with a home regimen consisting of oral cefixime and fusidic acid ocular gel, along with scheduled pediatric follow-up to monitor for recurrence or complications.

A consolidated CARE timeline of key events and interventions is shown in **Table 1**.

**Table 1:** CARE timeline summarizing clinical events, investigations, and treatments for two pediatric cases of staphylococcal scalded skin syndrome with early periocular involvement.

Day	Case 1 — events/findings		Case 1 — interventions		Case 2 — events/findings		Case 2 — interventions	
0	Periocular scaling; Oxford 3 OS; WBC 9.1×10^9^/L; Nikolsky +		IV nafcillin; fusidic acid gel; HOCl spray		Fever 37.8 °C; eyelid edema OU; WBC 8.5×10^9^/L; corneal staining − OU; Nikolsky +		IV teicoplanin; fusidic acid gel; HOCl spray	
1–2	Fever; generalized desquamation; cultures ETB+		Supportive care (fluids, analgesia, dressings)		Marked improvement in ocular/skin signs		Continue current therapy	
3	Ongoing desquamation		Switch to IV clindamycin (antitoxin)		MRSA confirmed (multi-site cultures)		Continue teicoplanin regimen	
4–6	Afebrile; WBC 5.9×10^9^/L; re-epithelialization		De-escalation; plan for oral therapy		Ongoing healing; re- epithelialization		Continue therapy (total 6 days)	
8	Discharge		Oral cephalexin + fusidic acid gel		Discharge		Oral cefixime + fusidic acid gel	

ETB, exfoliative toxin B; HOCl, hypochlorous-acid spray; MRSA, methicillin-resistant *Staphylococcus aureus*; OU, both eyes; OS, left eye; WBC, white blood cell count. Day 0 = emergency/initial hospital presentation. WBC expressed as ×10^9^/L. Day 0 denotes initial presentation. Systemic therapy (nafcillin→clindamycin in Case 1; teicoplanin in Case 2) was combined with topical ocular fusidic acid and periocular hypochlorous-acid spray. Both children showed rapid improvement and complete re- epithelialization without ocular sequelae. Abbreviations are defined below the table.

## Discussion

Staphylococcal scalded skin syndrome (SSSS) is primarily considered a dermatological emergency; however, as demonstrated by the present cases, ocular involvement can occur and may represent a clinically significant component of the disease. Although rare, ocular manifestations in SSSS warrant careful attention, as delayed recognition and treatment can lead to complications such as keratitis, conjunctivitis, or corneal ulceration, with potential long- term visual morbidity (5, 12). The Ocular adnexa exhibit distinct microbial tropisms with pathogen-specific immunologic responses (13–15), and may be particularly vulnerable to exfoliative toxins A/B because these serine proteases cleave desmoglein-1 (Dsg1), a key desmosomal cadherin enriched in the superficial epidermis of the eyelids and periocular skin. Relative paucity of compensatory desmoglein-3 in thin eyelid epidermis, together with local factors (friction from blinking/rubbing, moisture, and bacterial colonization), likely amplifies toxin-mediated epithelial fragility and explains early periocular signs (7, 16, 17). Conjunctival epithelium can also exhibit junctional perturbation from toxin exposure, manifesting as hyperemia and superficial keratopathy.

In both of our patients, periocular findings—including blepharitis, scaling, and conjunctival inflammation—were evident early in the disease course, coinciding with the onset of characteristic skin lesions and systemic symptoms such as malaise and fever. These observations are in line with prior reports suggesting that Staphylococcus aureus exfoliative toxins disseminate hematogenously from a localized infection site, producing widespread epidermal involvement, including the periocular skin. Notably, in Case 1, SSSS appeared to originate from a localized blepharitis of the left eye, underscoring that even seemingly minor ophthalmic infections (e.g., bacterial conjunctivitis or blepharitis) in susceptible hosts can serve as the initial focus for systemic toxin-mediated disease. Case 2 further expands this understanding, as ocular manifestations appeared very early and in conjunction with mucosal involvement, emphasizing that SSSS can initially mimic a primary ophthalmic condition, making prompt ophthalmological assessment essential for early diagnosis.

The management of ocular manifestations in SSSS is challenging because treatment must address both systemic and localized infections. In both cases, topical fusidic acid and hypochlorous acid were administered alongside systemic antibiotic therapy. Fusidic acid is known to be effective against S. aureus, including methicillin-resistant strains, and was instrumental in containing the localized periocular infection and preventing progression to corneal involvement (18). Hypochlorous acid, with its broad-spectrum antimicrobial and anti- inflammatory properties, provided additional periocular microbial control and supported wound healing (19).

Systemic antibiotic selection was tailored to disease severity and microbiological findings. In Case 1, initial IV nafcillin was switched to clindamycin to take advantage of its antitoxin effect, which is particularly relevant in toxin-mediated syndromes (11, 12, 18). In Case 2, the isolation of methicillin-resistant S. aureus guided the choice of teicoplanin, ensuring effective bacterial eradication. In both cases, early initiation of systemic therapy, combined with targeted ocular treatment, resulted in rapid symptom resolution and complete recovery without ocular sequelae.

Notably, the literature review identified a single case of SSSS exhibiting prominent ocular manifestations at disease onset. A 65-year-old healthy woman developed SSSS characterized by fever, purulent conjunctivitis, skin exfoliation, and a scarlatiniform rash. Staphylococcus aureus producing exfoliative toxin B was isolated from her eye discharge, nasopharynx, and skin lesions. This case is notable due to the rarity of SSSS in healthy adults, and the presence of purulent conjunctivitis as a key feature at onset, with the Staphylococcus aureus strain producing exfoliative toxin B but not TSST-1 or enterotoxin. The patient responded well to antibiotics and fluid supplementation (20).

This case report underscores the importance of considering SSSS in adults, regardless of their overall health status. The ocular involvement in this case is consistent with the two cases reported herein in terms of disease presentation, although it differs in terms of age, highlighting the potential for eyelid involvement in SSSS across different age groups.

These cases reinforce several important clinical lessons. First, periocular signs can precede or coincide with generalized SSSS manifestations, making ophthalmologists potential first- line identifiers of the condition. Second, SSSS may arise from localized ocular infections in pediatric patients, a presentation rarely documented in the literature. Third, effective management requires a multidisciplinary approach involving dermatology, pediatrics, and ophthalmology, with treatment tailored to microbial sensitivity and toxin inhibition. Finally, although pediatric SSSS generally carries a low mortality rate compared to adult cases, the potential for severe morbidity—including ocular damage—necessitates rapid recognition and intervention. However, we did not perform strain genotyping beyond exfoliative toxin profiling; future work incorporating molecular typing could clarify strain-specific ocular tropism and clinical severity.

## Conclusions

These two pediatric cases illustrate that ocular involvement in SSSS, although uncommon, can be an early and clinically relevant manifestation. In rare instances, as in our first case, staphylococcal blepharitis may serve as the initiating site of infection, subsequently evolving into full-blown SSSS. Early ophthalmologic examination was pivotal in both cases, enabling accurate diagnosis, microbiological confirmation, and prompt initiation of targeted systemic and local therapies.

The originality of our report lies in highlighting SSSS presentations in which ocular findings were either the first or an early manifestation, a feature that is underrepresented in current literature. This underscores the critical role of ophthalmologists in recognizing dermatologic emergencies and initiating multidisciplinary care. By increasing awareness of ocular signs in SSSS, we aim to promote earlier diagnosis, more effective intervention, and ultimately reduced morbidity in affected pediatric patients.

## Data Availability

The original contributions presented in the study are included in the article/supplementary material. Further inquiries can be directed to the corresponding authors.

## References

[1] Dajani AS . The scalded skin syndrome: relation to phage group II staphylococci. J Infect Dis. (1972) 125:548–51. doi: 10.1093/infdis/125.5.548, PMID: 4260083

[2] Patel NN Patel DN . Staphylococcal scalded skin syndrome. Am J Med. (2010) 123:505–7. doi: 10.1016/j.amjmed.2009.09.041, PMID: 20569752

[3] Napoli B D’Arpa N D’Amelio L Chimenti S Pileri D Accardo-Palumbo A . Staphylococcal scalded skin syndrome: Criteria for differential diagnosis from Lyell’s syndrome. Two cases in adult patients. Ann Burns Fire Disasters. (2006) 19:188–91., PMID: 21991049 PMC3188114

[4] Meshram GG Kaur N Hura KS . Staphylococcal scalded skin syndrome: A pediatric dermatology case report. SAGE Open Med Case Rep. (2018) 6:2050313X17750890. doi: 10.1177/2050313X17750890, PMID: 29326825 PMC5758955

[5] Blyth M Estela C Young AE . Severe staphylococcal scalded skin syndrome in children. Burns. (2008) 34:98–103. doi: 10.1016/j.burns.2007.02.006, PMID: 17644261

[6] Lamand V Dauwalder O Tristan A Casalegno JS Meugnier H Bes M . Epidemiological data of staphylococcal scalded skin syndrome in France from 1997 to 2007 and microbiological characteristics of Staphylococcus aureus associated strains. Clin Microbiol Infect. (2012) 18:E514–21. doi: 10.1111/1469-0691.12053, PMID: 23078129

[7] Amagai M Matsuyoshi N Wang ZH Andl C Stanley JR . Toxin in bullous impetigo and staphylococcal scalded-skin syndrome targets desmoglein 1. Nat Med. (2000) 6:1275–7. doi: 10.1038/81385, PMID: 11062541

[8] Handler MZ Schwartz RA . Staphylococcal scalded skin syndrome: diagnosis and management in children and adults. J Eur Acad Dermatol Venereol. (2014) 28:1418–23. doi: 10.1111/jdv.12541, PMID: 24841497

[9] Li MY Hua Y Wei GH Qiu L . Staphylococcal scalded skin syndrome in neonates: an 8-year retrospective study in a single institution. Pediatr Dermatol. (2013) 31:1–5. doi: 10.1111/pde.12114, PMID: 23557104

[10] Staiman A Hsu DY Silverberg JI . Epidemiology of staphylococcal scalded skin syndrome in US children. Br J Dermatol. (2018) 178:704–8. doi: 10.1111/bjd.16097, PMID: 29077993

[11] Patel GK Varma S Finlay AY . Staphylococcal scalded skin syndrome in healthy adults. Br J Dermatol. (2000) 142:1253–5. doi: 10.1046/j.1365-2133.2000.03571.x, PMID: 10848768

[12] Brazel M Desai A Are A Motaparthi K . Staphylococcal scalded skin syndrome and bullous impetigo. Med (Kaunas). (2021) 57:1157. doi: 10.3390/medicina57111157, PMID: 34833375 PMC8623226

[13] Strianese D Tranfa F Finelli M De Renzo A Staibano S Schiemer R . Hepatitis C virus infection in ocular adnexal lymphomas. Arch Ophthalmol. (2010) 128:1295–9. doi: 10.1001/archophthalmol.2010.233, PMID: 20937999

[14] Al-Sharif E Strianese D AlMadhi NH D’Aponte A dell’Omo R Di Benedetto R . Ocular tropism of coronavirus (CoVs): a comparison of the interaction between the animal-to-human transmitted coronaviruses (SARS-CoV-1, SARS-CoV-2, MERS-CoV, CoV-229E, NL63, OC43, HKU1) and the eye. Int Ophthalmol. (2021) 41:349–62. doi: 10.1007/s10792-020-01575-2, PMID: 32880786 PMC7471531

[15] Troisi M Del Prete S Troisi S Del Prete A Bellucci C Marasco D . The role of scanning electron microscopy in the evaluation of conjunctival microvilli as an early biomarker of ocular surface health: A literature review. J Clin Med. (2024) 13:7569. doi: 10.3390/jcm13247569, PMID: 39768491 PMC11727919

[16] Rolle CE Chen J Pastar I Cardenas TC Perez R Hower S . Keratinocytes produce IL-6 in response to desmoglein 1 cleavage by Staphylococcus aureus exfoliative toxin A. Immunol Res. (2013) 57:258–67. doi: 10.1007/s12026-013-8467-y, PMID: 24287883

[17] Amagai M Stanley JR . Desmoglein as a target in skin disease and beyond. J. Invest. Dermatol. (2012) 132:776–84. doi: 10.1038/jid.2011.390, PMID: 22189787 PMC3279627

[18] Yang T Wang J Cao J Zhang X Lai Y Li L . Antibiotic-resistant profile and thefactors affecting the intravenous antibiotic treatment course of generalized Staphylococcal Scalded Skin Syndrome: a retrospective study. Ital. J Pediatr. (2021) 47:169. doi: 10.1186/s13052-021-01120-6, PMID: 34362428 PMC8344213

[19] George SM Karanovic S Harrison DA Rani A Birnie AJ Bath-Hextall FJ . Interventions to reduce Staphylococcus aureus in the management of eczema. Cochrane Database Syst Rev. (2019) 2019:CD003871. doi: 10.1002/14651858.CD003871.pub3, PMID: 31684694 PMC6818407

[20] Oyake S Oh-I T Koga M . Staphylococcal scalded skin syndrome in a healthy adult. J Dermatol. (2001) 28:145–8. doi: 10.1111/j.1346-8138.2001.tb00108.x, PMID: 11349465

